# IMPROVING HAND FUNCTION AMONG OLDER ADULTS THROUGH AN INTERGENERATIONAL SERVICE-LEARNING PROGRAM

**DOI:** 10.1093/geroni/igad104.2779

**Published:** 2023-12-21

**Authors:** Rachel Logue Cook, Anna Schwartz, Courtney Vanderlaan, Susan Brown

**Affiliations:** University of Michigan, Ann Arbor, Michigan, United States; University of Michigan, Ann Arbor, Michigan, United States; Michigan Medicine, Ann Arbor, Michigan, United States; University of Michigan, Ann Arbor, Michigan, United States

## Abstract

Adequate hand function is essential for performing daily activities such as eating and dressing, but declines with age. Homebound older adults in particular are at greater risk for losing hand function necessary to remain independent. However, hand function in this population remains largely understudied. The purpose of this study is to determine whether Hands and Health at Home, an intergenerational upper limb-based intervention, improves hand function among homebound Meals on Wheels (MOW) clients. In this program, pre-health college students are trained to deliver a variety of hand strength, dexterity, and sensory exercises to a MOW client twice a week for 8 weeks. In addition, clients are instructed to complete exercises twice a week on their own. Measures of strength, dexterity, and sensation, as well as self-reported hand function are measured before and after the intervention. Preliminary results indicate MOW clients improved their grip strength an average of 42% in the dominant hand and 26% in the nondominant. Approximately 50% of clients improved dexterity (measured via the Purdue Pegboard) in their nondominant hand, and over 65% self-reported increased upper limb mobility. All clients and students valued this hands-on experience with several requesting continued involvement in the program. Results from Hands and Health at Home suggest this intergenerational approach to delivering hand therapy is an effective and mutually beneficial way to serve the needs of homebound older adults in the community and promote student interest in aging careers.

